# Gene Network Analysis of Glucose Linked Signaling Pathways and Their Role in Human Hepatocellular Carcinoma Cell Growth and Survival in HuH7 and HepG2 Cell Lines

**DOI:** 10.1155/2015/821761

**Published:** 2015-08-24

**Authors:** Emmanuelle Berger, Nathalie Vega, Michèle Weiss-Gayet, Alain Géloën

**Affiliations:** ^1^Lyon University, INSERM, UMR1060, INRA1397, CarMeN Laboratory, INSA, HCL, 69008 Lyon, France; ^2^Lyon University, Centre de Génétique et de Physiologie Moléculaire et Cellulaire (CGPhiMC), CNRS UMR5534, 69622 Lyon, France

## Abstract

Cancer progression may be affected by metabolism. In this study, we aimed to analyze the effect of glucose on the proliferation and/or survival of human hepatocellular carcinoma (HCC) cells. Human gene datasets regulated by glucose were compared to gene datasets either dysregulated in HCC or regulated by other signaling pathways. Significant numbers of common genes suggested putative involvement in transcriptional regulations by glucose. Real-time proliferation assays using high (4.5 g/L) *versus* low (1 g/L) glucose on two human HCC cell lines and specific inhibitors of selected pathways were used for experimental validations. High glucose promoted HuH7 cell proliferation but not that of HepG2 cell line. Gene network analyses suggest that gene transcription by glucose could be mediated at 92% through ChREBP in HepG2 cells, compared to 40% in either other human cells or rodent healthy liver, with alteration of LKB1 (serine/threonine kinase 11) and NOX (NADPH oxidases) signaling pathways and loss of transcriptional regulation of PPARGC1A (peroxisome-proliferator activated receptors gamma coactivator 1) target genes by high glucose. Both PPARA and PPARGC1A regulate transcription of genes commonly regulated by glycolysis, by the antidiabetic agent metformin and by NOX, suggesting their major interplay in the control of HCC progression.

## 1. Introduction

Liver is a central regulator of glucose homeostasis. Links between metabolism and tumorigenic processes have been mainly studied at the level of glucose uptake and release under metabolic stresses and diseases such as diabetes. Hyperglycemia itself may affect both glucose and lipid metabolism through the activation of stresses signaling pathways and the generation of reactive oxygen species (ROS) [1, 2]. Hyperglycemia may also regulate hexosamine pathways [3]. Glucose is also a major regulator of energy homeostasis through its transcriptional activity on insulin receptor [4], hormone sensitive lipase (HSL) [5], and genes relevant to high density lipids (HDL) metabolism [6]. Its transcriptional activity may also affect proinflammatory cytokines responsive genes involved in coagulation [7]. Moreover hyperglycemia could promote proliferation of hepatic stellate cells through mitogen-activated kinase (MAPK) activation and ROS production [8]. Thus alteration of liver functions greatly affects its responses to metabolic stress, and inversely alteration of energy homeostasis may alter liver cell function. The present study was designated to study the effect of high glucose on the proliferation and survival of hepatocellular carcinoma (HCC) cells and to identify the molecular mechanisms involved.

In HCC alterations of gene expression are mainly related to cell growth and maintenance, cell cycle, and cell proliferation as well as metabolism in humans [9–12]. Moreover HCC shares deregulation of translation proteins and transcription factors, such as hepatic nuclear factors 1A and 3b (HNF1 and HNF3b/FOXA2) or CCAAT/enhancer binding protein alpha (CEBPA) [13]. Cell signaling is mainly altered at the level of Wnt and MAPK signaling [14], that is, elevated activation of P42/44 (Erk1/2), which promotes cell growth and protects from toxic stresses [15]. Apoptosis and P38 MAPK activity are also reduced [16]. Abnormal activation of nuclear factor kappa B p65 subunit (NF*κ*B) promotes cell growth and survival and thus tumorigenic activity in HCC [17, 18]. Moreover, it is now well established that the energy sensor 5′-AMP-activated protein kinase (AMPK) plays a very important role in hepatic control of both proliferation and lipid metabolism [19, 20].

In a previous study, by using the real-time cell analysis (RTCA) system xCELLigence we identified major kinases required for cell survival and proliferation in two well characterized human HCC cell lines, HepG2 and HuH7 [12]. We have shown that protein kinase C (PKC), p42/44, Janus-kinase 1 (JAK1), NF*κ*B, and Jun-NH2 kinase (JNK) were required for HepG2 cell survival during 24 hours by treatment with specific inhibitors and serum removal. High glucose induces protein kinase C (PKC) activation, oxidative stress, and consequently reactive oxigen species (ROS) production [8, 21]. Resulting reduction of intracellular ATP affects protein kinase A (PKA) and adenylate cyclase activities [1], stimulates MAPK signaling, including Erk1/2 [8] and P38 MAPK (in HepG2 cells: [22]), PKC [2] and NF*κ*B signaling pathways [23], as well as NF*κ*B transcriptional activity (in HuH7 cells: [7]), and reduced basal activities of both AMPK and JNK pathways [24].

*In vitro* cell proliferation, survival and differentiation are highly dependent on experimental conditions such as cell density, stress, and nutrients. First of all we have determined time-dependant effects of cell density and serum deprivation on HepG2 and HuH7 cell proliferation and survival. Then we determined the modulatory effects of high (4,5 g/L)* versus* low glucose (1 g/L) concentrations. Using real-time proliferation assays, we found that the proliferation rate of HepG2 cells was independent of glucose concentration, opposite to that of HuH7 cells whose proliferation was reduced in low glucose. Using bioinformatic analyses of gene sets regulated (1) by glucose (2) differentially expressed in both cell lines in comparison to HCC and to healthy liver, we identified and validated on xCELLigence cell signaling pathways linked to the regulation of gene expression by glucose and dysregulated in HepG2 cells.

## 2. Experimental Procedures

### 2.1. Cell Culture, Treatment, and Analyses

The human hepatocarcinoma-derived cell lines HepG2 and HuH7 were provided from the European Collection of Cell Cultures (ECACC, Salisbury, UK). Cells were grown at 37°C in 5% CO_2_ in DMEM, glucose 4.5 g/L containing 10% fetal calf serum, complemented with streptomycin (100 *μ*g/mL) and penicillin (100 units/mL), and removed using trypsin 0.05% (PAA Laboratories, Les Mureaux, France). Both for plating and analysis, Scepter handheld automated cell counter (Millipore S.A.S., St Quentin-en-Yvelines, France) was used with 60 *μ*m tips on living cells in suspension, measured at least in triplicate and plated in the same culture media for one day before treatments. Cell proliferation and/or survival was monitored with the xCELLigence real-time cell analyser (RTCA) system (ACEA Biosciences Inc., San Diego, USA), which allows label-free monitoring changes of cell number, viability, morphology, and quality of cell attachment by measurement of cell-to-electrode responses of cells seeded in E96-well plates manufactured with integrated microelectronic sensor arrays. The results are represented as cell indexes (CI) impedance measurements or cell indexes normalized at time of treatment (i.e., CI at time *x* divided by CI at time of treatment) or slopes of linear curves after selected time of treatment. Since proliferation rate and cell index may vary from an experiment to another, data are representative experiments of at least three independent experiments and each condition was tested in at least 6 replicates. CI normalized to time of treatment depending on time are presented as mean values ± SEM with significant Student's *t*-test *p*-values *p* < 0.05. Cells were plated in 6-well plates for other experiments in the respect of cell plating density. For signaling pathway analyses, specific inhibitors were applied in either glucose 4.5 or 1 g/L serum-free media one day after plating. Drug concentrations were optimized for each compound according to dose-response analyses and half maximum inhibition of concentration IC50 (mean time-dependant IC50).

### 2.2. FACS Cell Cycle Analysis

Cells in suspension were fixed in ethanol 70% and then treated with 10 *μ*g/mL RNAse H (Promega, Charbonnières-les-Bains, France) in PBS during 1 hr before propidium iodine (Sigma Aldrich) was added (50 *μ*g/mL). Flow cytometric analysis of 5000 cells was performed on a FACSCanto II flow cytometer and data were recovered using the FACSDiva software v6.1.2 (BD Biosciences, Rungis, France). DNA content was determined using FlowJo software v8.8.6 (http://www.flowjo.com/).

### 2.3. Western Blots

After plating in 6-well plates, cells were lyzed in cell lysis buffer (EDTA 0.5 M, Na_3_VO_4_ 0,1 M, NaF 4%, DTT 0.1 M, Tris HCL 20 mM, KCL 2,7 mM, NaCl 138 mM, MgCl_2_ 1 mM, glycerol 5%, and 0,1% protease inhibitors). Protein contents were determined using Bradford assay and 30 *μ*g was loaded onto 7.5% SDS-Page electrophoresis, transferred onto PVFD membranes, and hybridized with 1/100 primary antibodies (Cell Signaling, Millipore S.A.S, Saint-Quentin-en-Yvelines, France) and 1/10 000 secondary rabbit antibody (Bio-Rad, Marnes-la-Coquette, France) according to standard procedures and revelation was performed with chemoluminescent ECL (Thermo Fisher Scientific, Perbio Science, Courtaboeuf, France). Western blots were performed in at least 3 independent experiments and scanned and quantification was performed with Image Quant software (GE Healthcare Life Sciences, Velizy-Villacoublay, France). Quantifications were performed as means of ratios of phosphorylated forms (first hybridization) compared to full isoforms (second hybridization after stripping) and Student's *t*-test *p*-value calculations.

### 2.4. Messenger RNA Quantification by Real-Time Quantitative PCR (RT-qPCR)

Total RNA purifications from HepG2 cells were performed according to standard protocol (Qiagen Quick prep mRNA, Qiagen, Courtaboeuf, France) including a DNase treatment. RNA integrity was assessed with the Agilent 2100 Bioanalyzer and RNA 6000 LabChip Kit (Agilent Technologies, Massy, France). First strand cDNAs were synthesized from 500 ng of total RNA in the presence of 100 U of Superscript (Invitrogen-Life Technologies, Eragny, France) and random hexamers and oligo-dT primers (Promega). Real-time quantitative PCR (RT-qPCR) was performed using ABsolute QPCR SYBR Green ROX Mix (Abgene, Courtaboeuf, France) with a Rotor-GeneTM 6000 system (Corbett Life Science, Cambridgeshire, UK). Levels of target mRNAs were normalized to hypoxanthine phosphoribosyltransferase 1 (HPRT1) expression measured in all samples by RT-qPCR. All quantifications were performed at least on three independent experiments and data are presented as means ± SEM. Gene names, references, functions, primers, and respective qPCR conditions have been published previously [12, 25].

### 2.5. Microarray Data Analyses

The sets of genes expressed in human normal liver, HCC and HepG2 cells, glucose, intracellular pathways, or transcription factors modulated by various stimuli were obtained exclusively with human cells, mostly cancer cell lines including HepG2 cells (Table 1). All gene sets originated either from published experiments or by analysis of datasets deposited in Gene Expression Omnibus (GEO) (http://www.ncbi.nlm.nih.gov/) using GEO2R web tool with significant *p*-values calculated according to the method of false discovery rate [26]. Liver set, that is, genes representative of healthy liver phenotype; HCC set, that is, genes dysregulated in HCC, and HepG2 set, that is, genes detected in HepG2 cells were raised from our previous published study [12]. These phenotypic gene sets were compared to published gene datasets regulated by either intracellular pathways or transcription factors listed in Supplementary Information (see Supplementary Information in Supplementary Material available online at http://dx.doi.org/10.1155/2015/821761). In gene set comparative analyses, significativity of enrichments were calculated using *z*-test with confidence level up to 95%.

## 3. Results

### 3.1. Comparative Analysis of HepG2 and HuH7 Cell Lines

**Figure 1:**
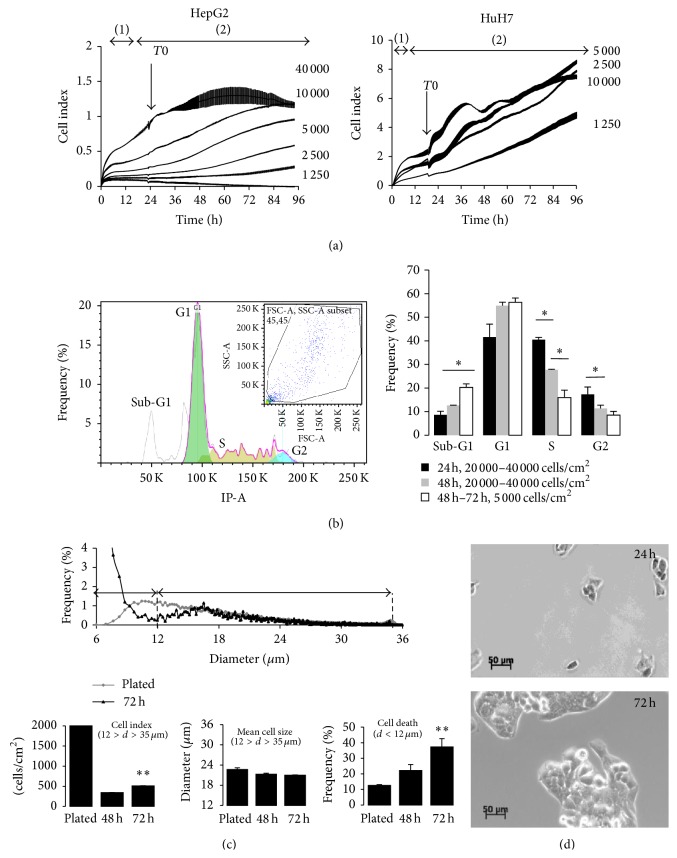
Cell density affects the rates of HepG2 and HuH7 cell growth, proliferation, and survival. (a) RTCA analysis represented by cell index (mean values ± SEM, *n* = 8): (1) adhesion phase and (2) proliferative phase. (b) Flow cytometry analysis of cell cycle by propidium iodine incorporation. Graphs illustrate both cell distribution (FSC-A, SSC-A subset) and their corresponding propidium iodine incorporation for 5000 cells, 72 hr. Significant increase in Sub-G1 (dying cells) was observed in low density plated cells, that is, 5000 cells/cm^2^ counteracted by reduced number of proliferative cells in S phase. In another way, proliferative cells were significantly reduced in high density plated cells, that is, 10000 to 20000 cells per well (mean values ± SD;  ^∗^Student's *t*-test *p*-value, *p* < 0.05). (c) Scepter cell count and cell size analysis of low density plated HepG2 cells (5000 cells/cm^2^) using 60 *μ*m tips. Cell index was reduced after 24–48 hr and then was increased within 72 hr. Cell size distribution was different in cells plated at high (80% confluency)* versus* low density plated cells 72 hr after serum removal. In low density plated cells, living cells were selected in a range of 12–35 *μ*m and smaller cells were considered as dying, dead cells, or cell fragments and thus they represent cell death. The fraction of cell death was significantly increased in low density plated cells and characterized by a significant reduction of mean size of living cells (mean values ± SD;  ^∗∗^Student's test *p*-value, *p* < 0.005). (d) Phase contrast micrographs (5000 cells/cm^2^, ×10) after 24 and 72 hours of cell culture. HepG2 cells were found to proliferate and grow in tridimensional groups.

In a previous study, we identified signaling pathways required for cell growth, proliferation, and/or survival of HepG2 cells, some of them have been validated in HuH7 cells, using real-time cell proliferation analysis on xCELLigence [12]. Cell growth, proliferation, and survival depend on cell type, density, and duration of treatments. In the present study, we aimed to analyze the effect of glucose on cell growth, proliferation, and survival. RTCA cell index represents cell surface occupancy, thus reflecting the sum of effects on growth, proliferation, and survival as well as on cell size and adhesion force. Several conventional methods were used to validate the experiments on xCELLigence; that is, Scepter cell counts reflect cell death, cell number, and cell size, and flow cytometry was used on selected conditions to analyze cell cycle. We found that cell density may affect the rate of proliferation and that mean size of proliferative cells increased in classical culture media (Figure 1). Thus cell culture conditions were optimized for each cell line in order to treat cells when RTCA cell index reached 0.5–1 for HepG2 and 0.5–1.5 for HuH7 cells one day after plating, that is, in linear phase of proliferation. Both cell lines present differences in their mode of proliferation, HepG2 cells in tridimensional space opposite to HuH7 cells which proliferate as monolayers and HepG2 cells which proliferate faster and their size is smaller than that of HuH7 cells (Table 2).

**Figure 2:**
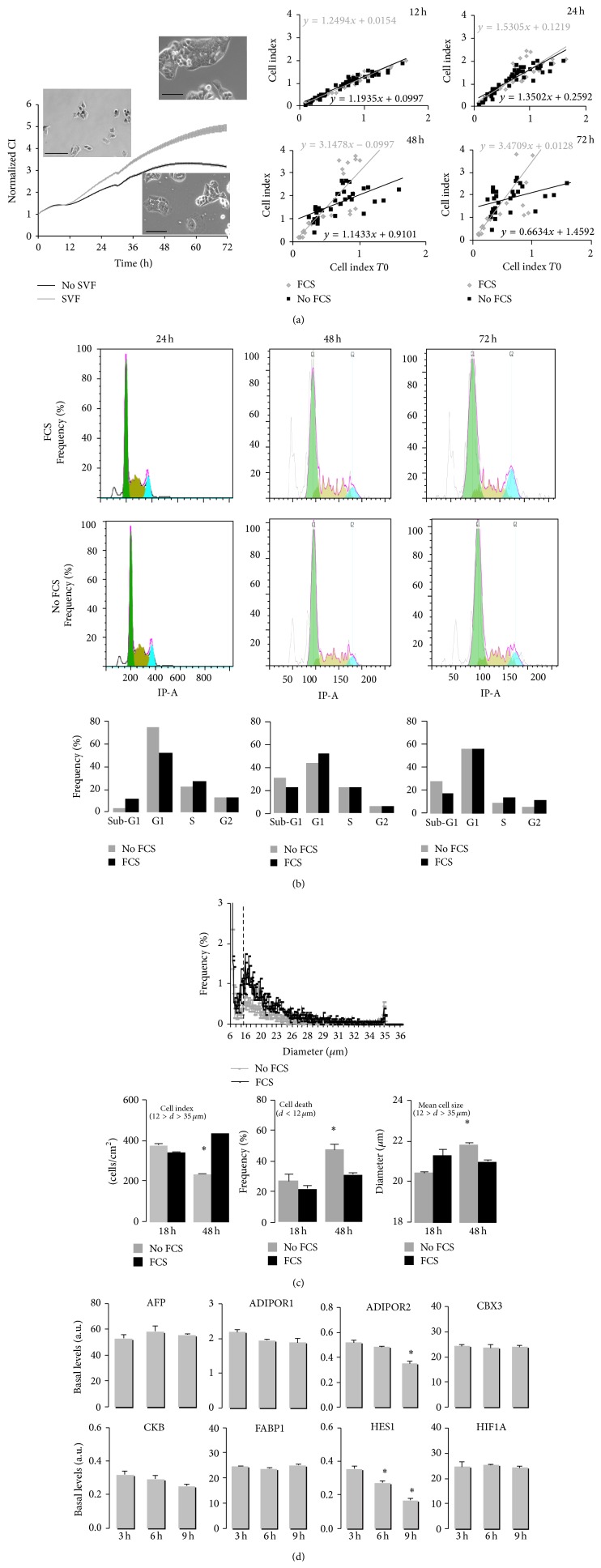
Serum depletion affects HepG2 cell growth and survival. (a) RTCA representative experiments (left panel) are presented by cell index normalized at time of media replacement (mean values ± SEM, *n* = 7). Micrographs were taken at time of treatment *T*0, 2 days and 4 days later, ×10 magnification, scale bar = 50 *μ*m. Right panels represent cell indexes obtained according to the values of cell index at time of media change (*T*0), 12 hr, 24 hr, 48 hr, or 74 hr after media replacement. Data were retrieved from 14 RTCA independent experiments. Tendency curves show that serum removal is sufficient to alter the proliferation and/or survival of HepG2 cells within 36 hr, whatever cell density is. (b) Representative flow cytometry analysis of HepG2 cell cycle by propidium iodine incorporation (IP-A). Graphs illustrate propidium iodine incorporation for 5000 cells per cm^2^; significant increase in Sub-G1 (dying cells) was observed in 48–72 hr serum depleted cells. (c) Scepter cell index and size analyses of low density plated HepG2 cells (5000 cells/cm^2^) after serum depletion. Living cells were selected in a range of 12–35 *μ*m and both their number and their size were reduced 48 hr after serum depletion. Inversely, the number of smaller cells representing cell death was significantly increased 48 hr after serum depletion and the mean size of living cells was significantly reduced (mean values ± SD, *n* = 3;  ^∗^Student's *t*-test *p*-value, *p* < 0.05). (d) Gene expression was performed by real-time qPCR analysis of genes representative of hepatic phenotype and function and/or dysregulated in hepatocellulocarcinoma. mRNA quantification was normalized to hypoxanthine phosphoribosyltransferase 1 HPRT1 (mean values ± SEM, *n* = 3 independent experiments,  ^∗^ANOVA test *p*-value < 0.05). AFP: alpha feto protein; AdipoR1/R2: adiponectin receptors 1 and 2; CBX3: chromobox protein 3; CKB: casein kinase B; FABP1: fatty acid binding protein 1; HES1: hairy enhancer of Split 1; HIF1A: hypoxia responsive gene 1.

In order to test specific effects of drugs on cell growth and/or survival, cells were plated in classical culture media and were treated one day later in serum-free media (Figure 2). Serum removal itself was found to reduce cell growth in both cell lines and in HepG2 cells, several genes representative of hepatic functions, that is, the adiponectin receptor 2 (AdipoR2) and the transcription factor Hairy Enhancer of Split 1 (HES1), were altered at transcriptional level 6 hours after serum removal.

### 3.2. Glucose Modulates Cell Growth and/or Survival of HuH7 Cells but Not That of HepG2 Cells

**Figure 3:**
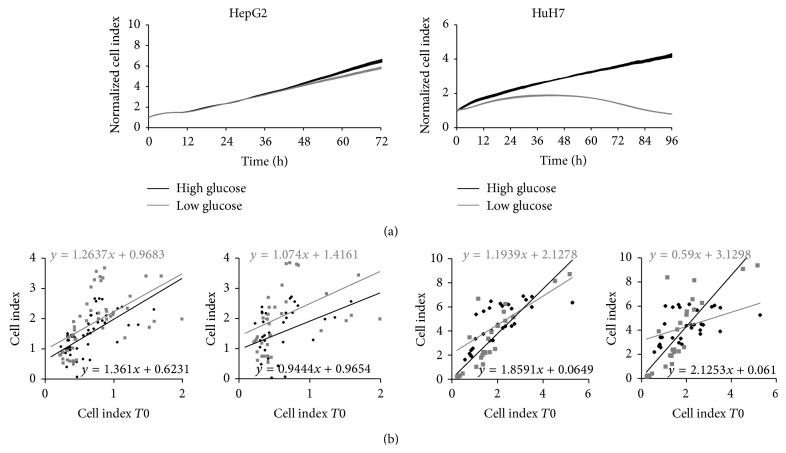
Glucose modulates HuH7 cell growth but does not affect HepG2 cells. Upper panels: RTCA representative experiment represented by cell index normalized at time of treatment, measured every 5 min. during 50 cycles and then every 15 min. Data are presented as mean values ± SEM (*n* = 8). Lower panels: analysis of cell indexes according to cell index at *T*0 (10 and 3 independent experiments for HepG2 and HuH7 cells, resp.) and corresponding tendency curves.

Classical culture media contain high glucose concentration (4.5 g/L) corresponding to systemic hyperglycemia. In low glucose concentrations corresponding to normoglycemia (1 g/L) the proliferation of HepG2 cells was not significantly affected although the rate of proliferation and the effect of glucose concentration were highly variable, especially at low or high cell densities (Figure 3). On the contrary, the proliferation rate of HuH7 cells was highly reduced in normoglycemic conditions. The results observed in experiments on RTCA system were confirmed by Scepter cell counts and cell cycle analyses on FACS (not shown).

### 3.3. Bioinformatic Analysis of Gene Networks Regulated by Glucose

**Figure 4:**
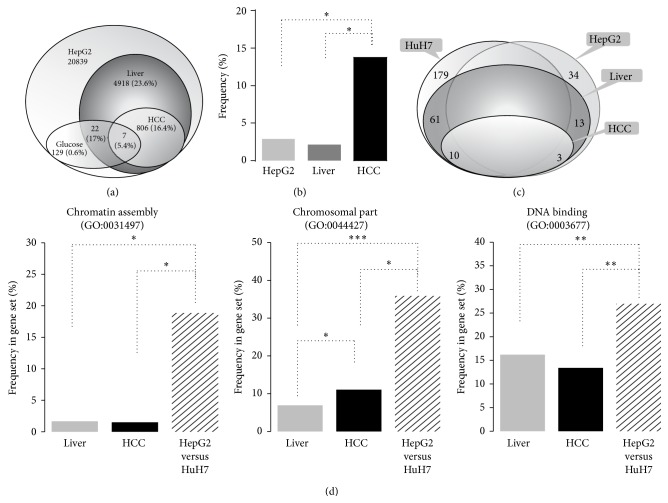
Gene sets representative of normal liver and dysregulated in hepatocellular carcinoma (HCC). Sets of genes detected in either HuH7 or HepG2 cells, specifically expressed in liver or dysregulated in HCC, or regulated by glucose, have been raised from the analysis of published datasets listed in Table 1. (a) Each set is represented by gene number and percentile of crossing set; for example, among the 129 genes regulated by glucose and detected in HepG2 cells (i.e., 0.6%), 22 belong to the liver phenotype set (i.e., 17% of them) and 7 are also dysregulated in HCC (i.e., 5.4% of them). (b) Frequency of genes regulated by high (4,5 g/L)* versus* low (1 g/L) glucose. Asterix represents significant overrepresentativity of genes regulated by glucose. (c) Gene sets differentially expressed in HepG2* versus* HuH7 cells, that is, 34 genes upregulated in HepG2 and 179 in HuH7 cell lines and representativity of liver and HCC genes in both sets. (d) Analysis of unique gene ontology (GO) biological process (left panel), cellular component (central panel), and molecular function (right panel) significantly overrepresented in the set of genes dysregulated in HepG2* versus* HuH7 cell line. The set of genes dysregulated in HepG2* versus* HuH7 (213 genes) was compared to sets of genes representing liver phenotype (4918 genes) or those dysregulated in hepatocellular carcinoma (806 genes) using FatiGO+ software (http://babelomics.bioinfo.cipf.es/).

Human gene sets obtained from published experiments using microarrays were retrieved in order to identify gene networks 1/regulated by glucose in healthy liver 2/dysregulated in HCC 3/differentially affected in HepG2 and HuH7 cell lines (Table 1). In a previous study, we have characterized a number of pathways dysregulated in HepG2 cells and linked to HCC [12]. A set of 339 genes regulated by high glucose were retrieved from studies on human cells (Table 1). A recent study identified a set of genes regulated either by glucose and by its major associated transcription factor carbohydrate-responsive element-binding protein (ChREBP) in HepG2 cells [27]. Among the 129 genes detected, 17% are linked to hepatic phenotype and 7 of them are dysregulated in HCC (Figure 4(a)).

**Figure 5:**
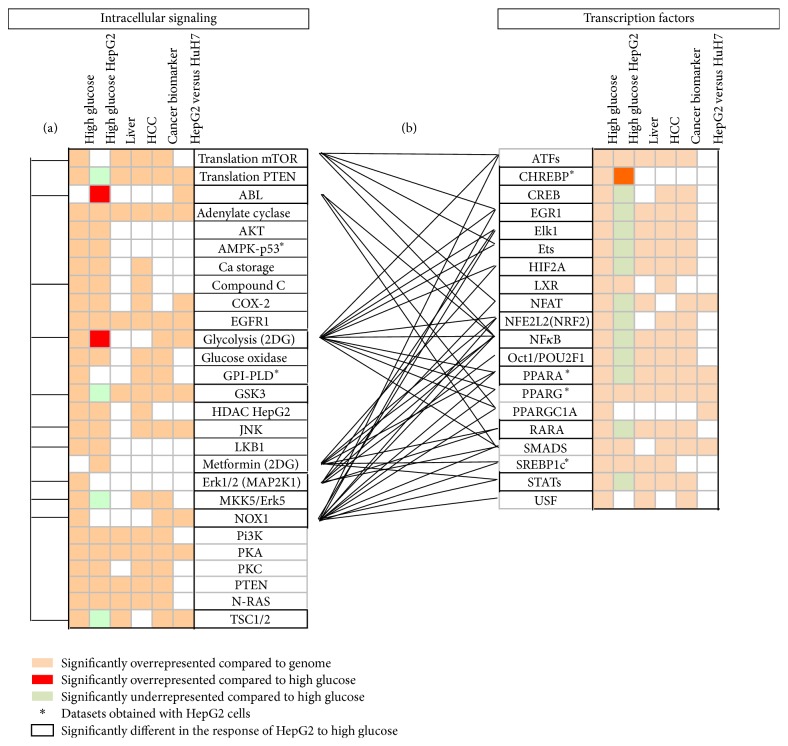
Transcriptional networks regulated by high glucose in human cells. The results were obtained by comparative analysis of datasets retrieved from the literature as defined in Table 1, that is, regulated by high glucose in human cells and more specifically in HepG2 cells, representative of either liver, hepatocellular carcinoma (HCC) phenotype, belonging to the list of cancer biomarkers or in the set of genes differentially detected in HepG2* versus* HuH7 cells. The left panel represents intracellular pathways with significantly overrepresented transcriptional targets in the set of genes regulated by high glucose, and the right panel presents the transcription factors whose target genes are significantly overrepresented in the set of genes regulated by high glucose. Left barrels (a) indicate significant overrepresentativity of signaling pathways differentially represented in the response of HepG2 cell lines to high glucose, and central barrels (b) represent transcription factors whose target genes are significantly overrepresented in these pathways. Significant differences in representativity were calculated for *z*-test confidence levels >95%. Abbreviations: HCC, hepatocellular carcinoma; others and full data are reported in Supplementary Information.

In a next step, we identified the molecular mechanisms involved in the control of proliferation by glycemia in HCC cells. Briefly, we retrieved published sets of human genes regulated by 48 intracellular signaling pathways and 52 transcription factors (Suppl. Information) and we compared their representativity in glucose responsive gene sets in humans and in HepG2 cells (representative of HCC cells) (Figure 5). We found that high glucose concentration regulated the transcription of genes common to 27 signaling pathways, including glycolysis, regulators of ROS production such as NADPH oxidase 1 (NOX1), glucose oxidase, LKB1/AMPK, and cyclooxygenase 2 (COX2), as well as second messenger signaling pathways (Pi3 Kinase Pi3K, protein kinases A and C…). Interestingly, most of these pathways are related to specific hepatic functions and they not only are subjected to altered expression in HCC but also belong to the set of cancer biomarkers.

### 3.4. Bioinformatic Analysis of Gene Networks Differentially Regulated in HepG2 versus HuH7 Cells

In a second study, we applied the bioinformatical approach to identify which differences from gene transcriptional networks could explain the differential sensitivities of HepG2 and HuH7 cell lines to glucose. Two sets of genes were retrieved from published experiments which lead us to select two sets of genes upregulated in HepG2 cells* versus* HuH7 (fold change > 1.4): (1) a comparative analysis to human cell lines [28] which may represent genes linked to cancer cells and (2) a comparison to several human liver cell lines [29], which may represent genes linked to hepatic function. We found 364 genes differentially expressed in HepG2* versus* HuH7 cells commonly found in both datasets, but only 213 genes were regulated in the same way (Figure 4(c); Suppl. Information); that is, 34 genes (16%) overexpressed in HepG2 in comparison to HuH7 cells; 74 of them were commonly found in liver gene sets (13 upregulated), 13 of them in HCC gene set. Using FatiGO+ software we found that this set of genes was significantly enriched in functions linked to properties of chromatin (Figure 4(d)). In the list of genes regulated by intracellular pathways, we found significant representativity of genes regulated by ABL, AMPKinase alpha 1 subunit (AMPKa1), calcium storage, cyclooxygenase 2 (COX2), glycosylphosphatidylinositol phospholipase D (GPI-PLD), JNK, PKA, RRM2B, Tuberous sclerosis 1 and 2 (TSC1/2), and regulator of translation PTEN. Among them, only ABL, GPI-PLD, glycogen-synthase 3 GSK3, TSC1/2, and regulation of translation by PTEN have been identified as dysregulated pathways in the response of HepG2 to high glucose (Figure 5). ABL and glycolysis are the pathways which are potentially overactivated in HepG2 upon high glucose. Both pathways were found to be significantly overrepresented in the set of genes dysregulated in HepG2* versus* HuH7 cells. Moreover in this set of 213 genes differentially expressed in HepG2* versus* HuH7 cells, 3 genes were found to be regulated by glucose, but only interleukin-6 receptor (IL6R) is linked to HCC.

### 3.5. Identification of Transcription Factors Linked to Glucose Signaling

A comparative analysis of datasets of genes regulated by high glucose to sets of genes regulated by 53 transcription factors retrieved from published microarray experiments on human cells (Table 2) suggests that high glucose may regulate transcription through at least 20 transcription factors (Figure 5) including glucose responsive ChREBP transcription factor; other factors involved in the transcriptional regulation of metabolism, such as sterol regulatory element binding protein 1 (SREBP1c), liver X receptor (LXR) or peroxisome-proliferator activated receptors (PPARs), and the PPARG coactivator 1 PPARGC1A, stress response pathways such as hypoxia-inducible factor 1a (HIF1a), Nuclear factor- (erythroid-derived 2)-like 2 (NFE2L2) and NF*κ*B, but also linked to proliferation, such as upstream transcription factors (USFs), cAMP responsive element binding protein 1 (CREB), early growth response factor (EGR1). This analysis suggests that, in HepG2 cells, ChREBP significantly regulates 92% of the genes also regulated by high glucose instead of 40% in human cells. In the set of genes differentially expressed in HepG2* versus* HuH7 cells we found overrepresentativity of genes regulated by PPARs, PPARGC1A, Smads, and nuclear factor of activated T-cells (NFAT), although the frequency of genes potentially regulated by high glucose in common with PPARA and PPARGC1A was increased and reduced, respectively, in HepG2 cells.

### 3.6. Experimental Validation of Signaling Pathways Linked to the Role of Glucose in Proliferation of HCC Cell Lines

**Figure 6:**
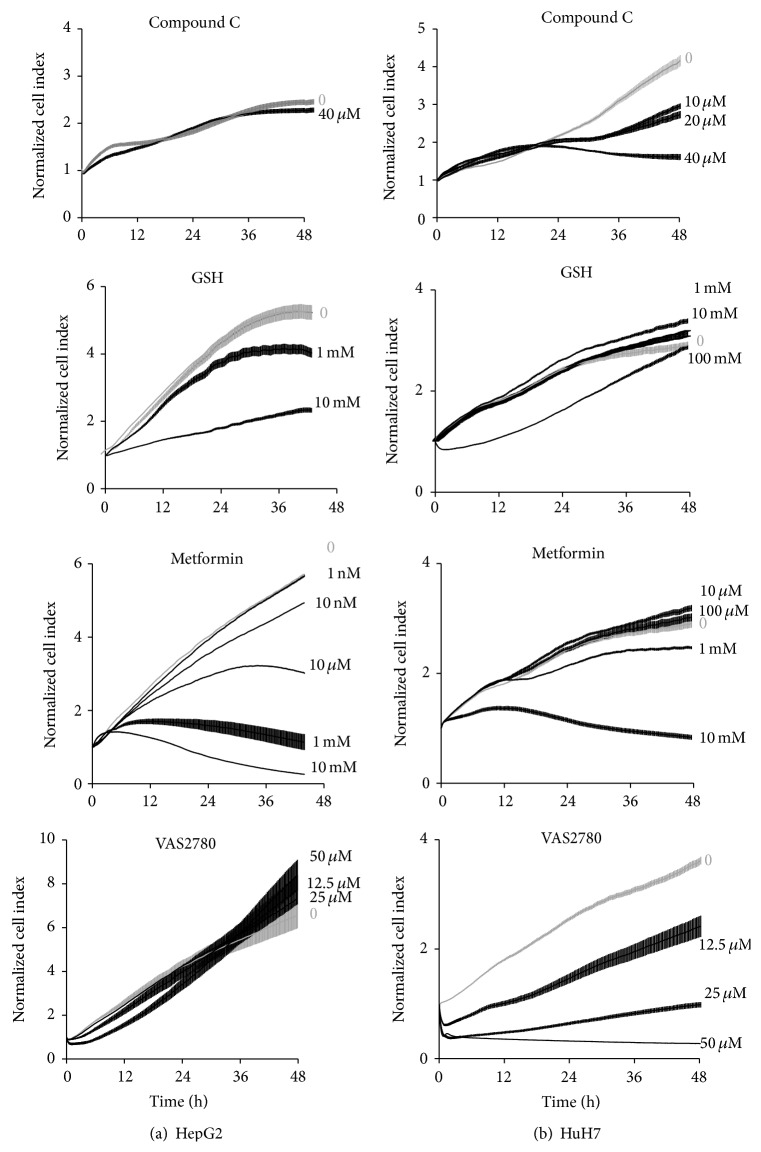
Signaling pathways with differential effect on proliferation in HepG2 cells* versus* HuH7 cells. Real-time proliferation assays on xCELLigence were performed in either low or high glucose serum-free media in presence of either LKB1/AMPK pathway inhibitor Compound C, mitochondrial apoptosis inhibitor reduced glutathione (GSH), LKB1 activator metformin, or NADPH oxidases inhibitor VAS2870. Dose-dependent responses were analyzed in high glucose-serum free media. Representative RTCA experiments are presented as cell index normalized at time of treatment according to time and slopes (mean values ± SEM, *n* = 8) in HepG2 (left panels) and HuH7 cell lines (right panel).

In a previous study [12] we used real-time proliferation assay using xCELLigence system to identify signaling pathways involved in HepG2 cell growth and/or survival and dysregulated in HCC, in which inactivation by specific inhibitors leads to cell growth arrest and/or cell death one day after treatment. The effect of glucose on HepG2 and HuH7 cell growth was observed in extended times, that is, 48–72 hours in serum-free media, and selected signaling pathways were analyzed in such conditions. First of all we analyzed pathways that merged from bioinformatic analyses, and we observed that proliferation through JNK and Pi3 kinase pathways were altered in a dose-dependent manner in both HuH7 and HepG2 cells (Table 3). The main differences between HuH7 and HepG2 cells were observed at the level of LKB1, AMPK, and mitochondrial stress pathways (Figure 6): HuH7 but not HepG2 cells were sensitive to Compound C, a specific inhibitor of AMPK, and to VAS2780, a specific inhibitor of NOX, opposite to a higher sensitivity of HepG2 cells to reduced glutathione and to metformin. Other kinases known to be regulated by glucose were tested: inhibition of P38MAPK slightly affected cell growth and proliferation only in high glucose concentrations in HepG2 cells. We have previously shown that inhibition of either p42/44 or PKC leads to cell death and that of NF*κ*B leads to cell growth arrest [12]. Similar results were obtained on HuH7 cells.

**Figure 7:**
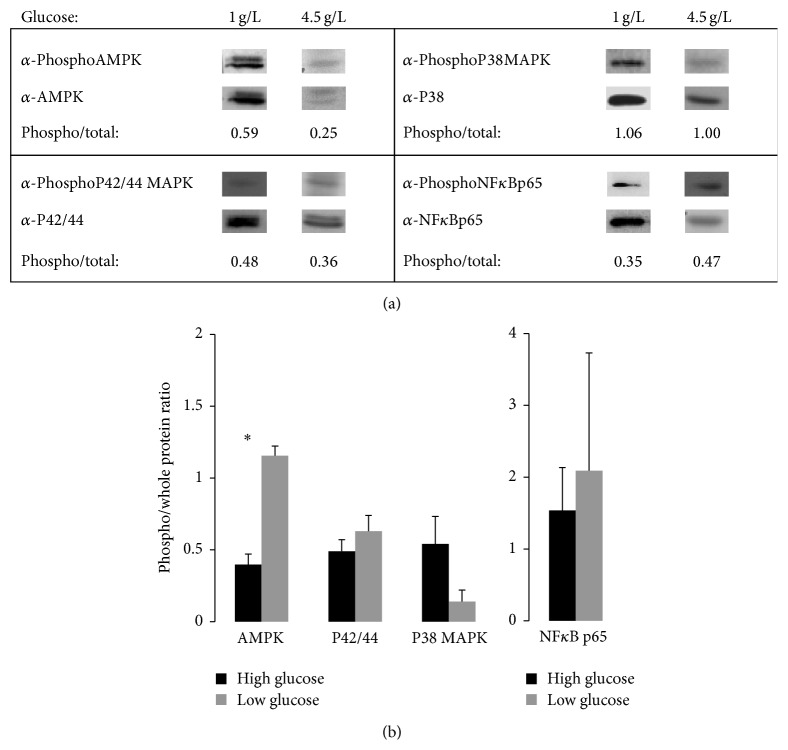
Basal activities of AMPK, P42/44, P38 MAPK, and NF*κ*B p65 in HepG2 cells grown in either 1 (Low) or 4,5 g/L (High) glucose serum-free media during 30 mn. Western blots were performed with the same protein extracts, and blots were hybridized first with antiphosphorylated kinase antibody (*α*-Phospho), stripped, and then hybridized with antibody raised against whole kinase form (*α*-). (a) Representative blots. (b) Ratio of electrophoretic bands detected by phosphoprotein* versus* whole protein antibodies (mean values ± SD). Asterix indicates significant difference in high* versus* low glucose treatment.  ^∗^Student's *t*-test *p*-value, *p* < 0.05 (3 independent experiments).

The effects of glucose on kinase activation in HepG2 cells were analyzed by western blot (Figure 7), showing high basal activated forms of P42/44 and low basal activated forms for P38MAPK. AMPK was characterized by high basal activity and its partial reduction in high glucose concentrations. NF*κ*B p65 subunit was highly activated independently of glucose concentration but with high variability in high glucose concentration.

### 3.7. Glucose Regulates Gene Transcription of Metabolic and Proliferative Target Genes in HepG2 Cells

**Figure 8:**
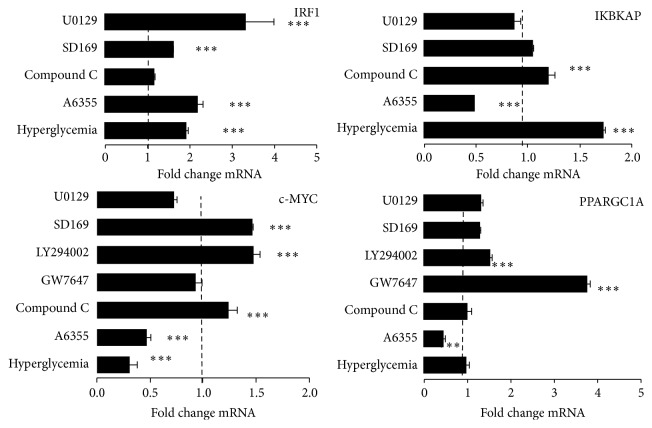
Regulation of gene transcription by glucose in HepG2 cells measured by RT-qPCR. Analysis of specific pathways in low glucose with P42/44 inhibitor A6355, 90 *μ*M; AMPK inhibitor Compound C, 40 *μ*M; Pi3 kinase inhibitor LY294002 10 *μ*M, P38MAPK inhibitor SD169 10 *μ*M; and Mek1/2 inhibitor U0129 10 *μ*M and PPARA activator GW7647 1 *μ*M. Cells were treated in serum-free media during 5 hours in at least three independent experiments. Data are presented as mean fold change mRNA of treated sample* versus* vehicle, normalized to HPRT1 (hypoxanthine phosphoribosyltransferase 1) ± SEM. Significant effects were selected for Student's *t*-test  *p*-values, ^∗∗∗^ *p* < 0.001.

We have previously selected a number of genes either representative of healthy liver and dysregulated in hepatocellular carcinoma [12]. HepG2 cells were treated by either high or low glucose serum-free media during 5 hours (Table 4). Only 3 genes were upregulated by high glucose, that is, apolipoprotein C3 (APOC3), inhibitor of kappa light polypeptide gene enhancer in B cells (IKBKAP), and interferon-responsive factor 1 (IRF1). Several genes were analyzed in low glucose concentration in presence of specific modulators for pathways at concentrations determined previously. In low glucose media, that is, with activated AMPK, the specific AMPK inhibitor Compound C did not affect the transcript levels of IRF1 (Figure 8) and APOC3 (not shown). IRF1 gene transcription was increased in the presence of either P42/44, Mek1/2, or P38MAPK inhibitors. The level of mRNA for IKBKAP was not affected by either P38MAPK or Mek1/2 but was reduced in the presence of P42/44 inhibitor. Among the 15 genes whose transcription was reduced by high glucose, the most altered gene transcripts were transcription factors forkhead box O1 (FOXO1A), linked to metabolism, EGR1, and v-myc myelocytomatosis viral oncogene homolog (MYC) which both play major roles in cell growth, proliferation, and tumorigenesis. We found that AMPK inhibition did not significantly affect MYC mRNA levels although P38MAPK and Pi3K act as repressors. PPARGC1A gene is a classical transcriptional target of AMPK, and we found that, in HepG2 cells, high glucose and AMPK inactivation did not modulate its level of transcription, although P42/44 and PPARA were activators and Pi3K was an inhibitor of PPARC1A gene transcription.

## 4. Discussion

The emerging concept that tumorigenic processes largely depend on metabolism opens new challenges and needs reevaluation of old concepts using integrative studies. In this context, nutrients should be explored to identify their potential activities as pro- or anticancerous signals. Liver is the central regulator of glucose homeostasis. High glucose concentration induces metabolic changes in liver, including not only glucose and fatty acid metabolism but also stress [30]. We have selected two human cell lines which are representative of human HCC, HuH7, and HepG2 cell lines, in order to study how glucose may affect their growth, proliferation, and/or survival. HuH7 cell line is still able to differentiate in hepatocytes at confluency, opposite to the highly proliferative HCC cell line HepG2. Real-time experiment assays confirmed that the proliferation rate was higher for HepG2 than HuH7 cells. HepG2 cells were found to be highly sensitive to stress, because they were more sensitive to serum deprivation than HuH7 cells and presented variable NF*κ*B activations in high glucose concentrations (Figure 7). Among the numerous differences between these two human cell lines, there are the following: their methylation status [31], the highest expression of hepatocyte differentiating transcription factors CEBPA and HNF4A in HuH7 in comparison to HepG2 cells [32], and lack of the detoxifying enzyme COX2 in HepG2 cells [33] despite the fact that this enzyme is able to induce cell death in HCC cells [34]. In addition, previous studies have shown that cytochrome CYP450 genes are less expressed in HepG2 than in hepatocytes [35–37]. In this study, we show that high glucose concentration can promote cancer cell proliferation on the HCC cell line HuH7, but without any significant effect on HepG2 cells. We took advantage of this result to identify glucose-mediated proliferative pathways.

Through bioinformatic analyses we selected a set of 213 genes differentially expressed in both cell lines; 84% of them are downregulated in HepG2 cells, and interestingly we found that IL6R gene transcription was overinduced in HepG2 cells although it is downregulated by glucose [27]. In liver IL6/JAK/STAT3 pathway, the major activator of acute-phase proteins [38] regulates gluconeogenesis [39] and induces growth arrest in HCC through regulation of cyclin-dependant kinases and CDKN1A gene expression [40]. IL6/Erk1/2 pathway activates cell proliferation through transcriptional activation of immediate-early responsive genes such as FOS, JUN, and EGR1 in rat hepatocytes [41]. Alteration of IL6R gene transcription is correlated with tumor grade in HCC [38], and in the initial processus of hepatocellular transformation, this receptor participates in hepatocellular transformation [42]. Thus, it is considered as an additional marker to AFP for HCC diagnosis [43]. In HepG2 cells P42/44 and STAT3 are constitutively activated [44]; thus our results point out a major role of IL6 pathway in the progress of tumorigenesis in liver under high glucose.

Through bioinformatic analyses of gene set data, we found that glucose regulated gene transcription through NOX and several protein kinase pathways. Intracellular pathways modulated by extracellular glucose involve PKC, MAP kinases, and glucokinase. We found that, in HepG2 cells, crosstalk of glucose signaling with those of fatty acids and insulin, including intracellular transducers JNK, P38MAPK, Rac1, PTEN, and mTOR, is highly affected and that JNK, mTOR, and TSC1/2 were particularly affected in comparison to HuH7 cells. Through real-time monitoring of cell proliferation and/or survival of both cell lines cultured in high* versus* low glucose conditions, we found that glucose activated HuH7 cell growth, but not that of HepG2 cells, through NOX and LKB1 pathways. LKB1 regulates hepatic glucose homeostasis through modulation of AMPK/TORC2 activity and consequently transcriptional regulation of PPARGC1A which in turn drives neoglucogenesis [45]. High glucose inactivates AMPK and is known to induce oxidative stress and ROS production through PKC, NF*κ*B, and NOX activation and AMPK itself regulates the activity and the transcription of several NOX [45, 46]. We found that AMPK activity was reduced in HepG2 cells cultured in high glucose concentrations. This results support the hypothesis that high glucose may promote cell proliferation in HCC cells by inactivation of AMPK and increased activity of NOX.

In addition, inhibition of NOX has been previously shown to inhibit EGFR pathway and TGF beta induced apoptosis in liver cells via P42/44 and Akt and to inhibit cell growth without apoptosis in HepG2 cells [47]. In HepG2 cells, P42/44 is constitutively active and thus may escape from AMPK and NOX regulatory activities on ROS–mediated apoptosis. AMPK level of expression itself is reduced in HepG2 cells [3]. This phenomenon may be increased in absence of regulators of ROS production, such as COX2 and GSTP1 (mRNA not detected in our experiments, data not shown) which both regulate JNK to induce apoptosis [48, 49]. This result is in accordance with the high level of stress oxidative responsive genes in HepG2 cells [50], thus conferring survival advantage to HepG2 cells [51].

We have identified putative glucose-linked transcription factors at the crosstalk between metabolism and cell growth, such as MYC, PPARs, LXR, or FKHR, as well as the glucose-responsive transcription factor ChREBP, which modulates the expression of 92% of the genes commonly regulated by high glucose in HepG2 cells instead of 40% in human cells. This result was not specifically linked to liver phenotype, as in rodents, we found that Mlx/ChrEBP similarly regulates 42% of hepatic genes regulated by glucose [46]. Taken together with the inactivation of AMPK by high glucose in HepG2 cells, this analysis suggests that the classical glucokinase/ChrEBP pathway involved in glucose-dependant gene transcription is normally regulated by AMPK in HepG2 cells [52]. AMPK regulates PPARGC1A, a central coactivator of transcription factors involved in mitochondrial biogenesis and metabolic pathways linked to the faster response in liver, including neoglucogenesis [53]. This regulation involves the modulation of both PPARGC1A activity through histone deacetylase/NAD+/SIRT1 pathway and crosstalk with its transcriptional regulation by Pi3k/mTORC2/CREB pathway [54]. We found that HepG2 cells were 100 more sensitive to both the antiproliferative activity of metformin and reduced glutathione (Figure 5). Metformin induces activation of AMPK/SIRT1 and reduces p53 abundance in HepG2 cells in high glucose media [55], and p53 itself is less expressed in HepG2 than in HuH7 cells [56]. Such a difference may explain the differential sensitivity to metformin. Moreover, SIRT1 functionally interacts with PPARGC1A [57]. In our experiments, we found that PPARGC1A mRNA was not regulated by high glucose or inactivation of AMPK basal activity although it was repressed by Pi3K and PKA and activated by Erk1/2 and PPARA. Gene transcription analysis of PPARGC1A associated transcription factor pathways did not revealed any dysregulation of its activity, as illustrated by APOC3, target gene of ERRalpha [58], CREBBP regulated by Foxo1A [59], and HNF4A and APOC3 by HNF4A [60–62], or AdipoR2, CEBPA, and APOC3, which are target genes of PPARA and PPARG [12, 63]. These results indicate that gene transcription was not significantly altered at the level of PPARGC1A linked pathways.

Examination of microarray datasets supports the hypothesis that PPARGC1A is less abundant in HepG2 than in HuH7 cells and moreover its level of expression is frequently downregulated in HCC [64], including HepG2 cells, and is essential for proper hepatic gene transcription and differentiation [65]. We found that PKC, Pi3K, P38MAPK (inhibitors), and Mek/Erk (activator) signaling pathways were preserved in HepG2 cells although AMPK pathway failed to regulate MYC gene expression in HepG2 cells, possibly through alterations of both STAT3, constitutively activated in HepG2 cells [44], and STAT-responsive element in the promoter of MYC [66]. Both PPARGC1A and MYC gene transcription are also regulatable by CREB via opposite effects of Pi3k/mTORC2 and AMPK; however, this transcriptional regulation is preserved in HepG2 cells [67]. These results confirm previous published data showing that high glucose inhibits MYC transcription in these cells [68].

In conclusion, high glucose concentrations differentially modulate cell growth and survival in both HCC cell lines without significant alteration of the pathways linked to neoglucogenesis. Glycolysis, glutaminolysis, and oxidative phosphorylation are the main sources of energy, that is, NADH, NADPH, and ATP [69]. We found that the LKB1 pathway is a central regulator of the proliferation induced by high glucose in HCC cells. In HepG2 cell line high basal PKC and P42/44 activities are linked to loss of control on cell growth by glucose through the metabolic pathway LKB1/AMPK/NOX.

## Supplementary Material

Suppl Information 1 : Gene datasets regulated by intracellular pathways (left panel) and transcrition factors (right panel).
Suppl Information 2: Hepatic genes differentially expressed in Hepg2 *versus* HuH7 cells (red: up-regulated, green: down-regulated). Genes present in hepatocellularcarcinoma gene sets are in yellow. IL6R is a unique gene regulated by glucose (27).

## Figures and Tables

**Table 1:** Datasets retrieved from the literature and used to build phenotype sets.

	Data source	Genes number	Genes with intracellular pathway	Genes with transcription factor
			*n* (%)	*n* (%)
Genome	FatiGo+ database	31524		

Detected in HepG2	Microarray data analysis [12]	20839	11705 (56%)	10477 (50%)

Liver	Microarray data analysis [12]	4918	1631 (33%)	1676 (34%)

HCC	Microarray data analysis [12]	806	516 (64%)	560 (69%)

Cancer biomarkers	Genes representing cancer biomarkers [70]	1262	755 (60%)	815 (65%)

Deregulated in HepG2 versus HuH7	Common genes from:	213	146 (69%)	132 (62%)
	(i) Comparative analysis of human cell lines [28]			
	(ii) Comparative analysis of liver cell lines [29]			

High glucose	(i) Common genes from:	447	307 (69%)	336 (75%)
	(a) Aortic cells [71]			
	(b) Leukocytes (GSE32909)			
	(c) Vascular smooth muscle (GSE17556)			
	(ii) HepG2 [27]	129	98 (76%)	124 (96%)

**Table 2:** Human hepatocellular carcinoma cell lines characteristics.

	HepG2	HuH7
Number of plated cells/cm^2^	2500–5000	1250–2500
Proliferation in space	Tridimensional	Monolayer
Doubling time (xCELLigence)	8 hr	12 hr
Mean cell size (Scepter)	20.9 *μ*M	21.9 *μ*M

**Table 3:** Pathways tested on real-time experiments.

Pathway	Drug activator (A)/inhibitor (I)	Highest dose tested	Time of significant effect	HepG2		HuH7	
				Effect	IC50 (*μ*M)	Effect	IC50 (*μ*M)
AMPK	AICAR (A)	2 mM	24 hr	−	3 10^−3^	−	1.1 10^−3^
	Compound C (I)	40 *μ*M	24 hr	No effect		−	3.3 10^−5^
JAK1	Butein (I)	50 *μ*M	24 hr	−	2 10^−5^	−	Nd
JNK	SP600125 (I)	100 *μ*M	24 hr	−	1.8 10^−5^	−	2.2 10^−5^
LKB1	Oligomycin (A)	2 mM	48 hr	−	3 10^−7^	−	2.5 10^−7^
	Metformin (A)	10 mM	24 hr	−	7 10^−6^	−	8 10^−4^
Mek1/2	U0126 (I)	250 *μ*M	1 hr	−	1.1 10^−5^	−	Nd
mTOR	Rapamycin (I)	200 nM	48 hr	No effect		No effect	
Mitochondrial apoptosis	GSH (A)	10 *μ*M	24 hr	−	10^−6^	−	1.3 10^−4^
NF*κ*B p65	Wedelolactone (I)	10 *μ*M	24 hr	−	0.8 10^−5^	−	10^−5^
NOX	VAS2780 (I)	50 *μ*M	1 hr	−	5 10^−5^	−	1.8 10^−5^
P38MAPK	SD169 (I)	300 *μ*M	24 hr	+	9.4 10^−5^	No effect	
P42/44	A6355 (I)	90 *μ*M	1 hr	−	−	−	−
PKA	KT5720 (I)	20 *μ*M	48 hr	+	1.1 10^−4^	+	Nd
PKC	P3115 (I)	50 *μ*M	1 hr	5 10^−6^	−	−	Nd
Pi3K	LY294002 (I)	50 *μ*M	48 hr	−	3.3 10^−5^	−	3 10^−5^
PPARA	GW6471 (I)	1 mM	1 hr	−	5.7 10^−4^	−	4 10^−4^

AMPK, 5′-AMP-activated protein kinase; AICAR: 5-aminoimidazole-4-carboxamide ribonucléotide; GSH: reduced glutathione; JAK1: Janus kinase 1; JNK: Jun-NH2 kinase; LKB1: serine/threonine kinase 11; NF*κ*Bp65: nuclear factor kappa B p65 subunit; NOX: NADPH oxidases; P38MAPK: P38 mitogen-activated kinase; Pi3K: Pi3 kinase; PKA: protein kinase AMPc-dependant; PKC, protein kinase C; and PPARA, peroxisome-proliferator activated receptor alpha.

**Table 4:** Regulation of gene transcription by glucose in HepG2 cells measured by RT-qPCR. Levels of gene expression in high (4.5 g/L) versus low (1 g/L) glucose concentrations.

Symbol	Gene name	Gene ID	Fold change
Upregulated			
APOC3	Apolipoprotein C-III	345	2.83 ± 0.08
IRF1	Interferon regulatory factor 1	3659	1.92 ± 0.05
IKBKAP	Inhibitor of kappa light polypeptide gene enhancer in B cells, kinase complex-associated protein	8518	1.73 ± 0.03
Down-regulated			
FOXO1A	Forkhead box O1	2308	−9.09 ± 0.02
EGR1	Early growth response 1	1958	−8.33 ± 0.06
CREBBP	CREB binding protein (Rubinstein-Taybi syndrome)	1387	−3.23 ± 0.01
MYC	v-myc myelocytomatosis viral oncogene homolog (avian)	4609	−3.23 ± 0.07
ADIPOR2	Adiponectin receptor 2	79602	−3.03 ± 0.20
BHLHB2	Basic helix-loop-helix domain containing, class B, 2	8552	−2.86 ± 0.06
CDKN1B	Cyclin-dependent kinase inhibitor 1B (p27, Kip1)	6043	−2.27 ± 0.01
SOD2	Superoxide dismutase 2, mitochondrial	6648	−2.04 ± 0.20
MAP4K4	Mitogen-activated protein kinase kinase kinase kinase 4	9448	−1.82 ± 0.06
CEBPB	CCAAT/enhancer binding protein (C/EBP), beta	1051	−1.79 ± 0.02
FOXA2/HNF3b	Forkhead box A	3170	−1.67 ± 0.09
TGFB1	Transforming growth factor, beta 1	7040	−1.61 ± 0.01
TNFRSF1A	Tumor necrosis factor receptor superfamily, member 1A	7132	−1.49 ± 0.01
HES1	Hairy and enhancer of split 1, (drosophila)	3280	−1.45 ± 0.00
HNF4A	Hepatocyte nuclear factor 4, alpha	3172	−1.43 ± 0.05
Not regulated			
ADIPOR1	Adiponectin receptor 1	51094	
AFP	Alpha fetoprotein	174	
CAT	Catalase	847	
CEBPA	CCAAT/enhancer binding protein (C/EBP), alpha	1050	
FABP1	Fatty acid binding protein 1, liver	2168	
FOXM1/HNF3	Forkhead box M1	2305	
HK2	Hexokinase 2	3099	
LDLR	Low density lipoprotein receptor	3949	
PPARG	Peroxisome proliferator-activated receptor gamma	5468	
PPARGC1A	Peroxisome proliferator-activated receptor gamma, coactivator 1 alpha	10891	
TBP	TATA box binding protein	6908	

See Figure 8.

## Abbreviations

ABL: c-ABL oncogene 1, nonreceptor tyrosine kinase
ADIPOR2: Adiponectin receptors 2
AMPK: 5′-AMP-activated protein kinase
APOC3: Apolipoprotein C-III
BHLHB2: Basic helix-loop-helix family, member e40
ATP: Adenosine triphosphate
CAR: Constitutive androstane nuclear receptor
CDKN1A: Cycle dependant kinase inhibitor A1 (p21, Cip1)
CEBP (A-G): CCAAT/enhancer binding protein (alpha and gamma subunits)
ChREBP: Mlx interacting protein-like
COX2: Cyclooxygenase 2
CREB: cAMP responsive element binding protein 1
CREBBP: CREB binding protein
CYP450: Cytochromes P450
DMEM: Dulbecco's minimum essential media
E2F: Elongation factors 2
EGR1: Early growth response factor
FCS: Fetal calf serum
FOS: BJ murine osteosarcoma viral oncogene homolog
FOXO1A (FKHR): Forkhead box O1
ERRalpha: Estrogen receptor alpha
GO: Gene ontology
GSK3: Glycogen synthase kinase 3
FOXA2 (HNF3b): Forkhead transcription factor A2
GPI-PLD: Glycosylphosphatidylinositol phospholipase D
GSTP1: Glutathione S-transferase pi 1
HCC: Hepatocellular carcinoma
HNFs: Hepatocyte nuclear factors (1–4)
HPRT1: Hypoxanthine phosphoribosyltransferase 1
IKBKAP: Inhibitor of kappa light polypeptide gene enhancer in B-cells, kinase complex-associated protein
IL6: Interleukin-6
IL6R: Interleukin-6 receptor
IRF1: Interferon-responsive factor 1
JAK: Janus kinase
JNK: Jun-NH2 kinase
LKB1 (STK11): Serine/threonine kinase 11
LXR: Liver X nuclear receptor
MAPK: Mitogen activated protein kinase
mTOR (C1 and C2): Mechanistic target of rapamycin (serine/threonine kinase)
MYC: v-myc myelocytomatosis viral oncogene homolog
NFAT: Nuclear factor of activated T-cells, cytoplasmic, calcineurin-dependent 1
NFE2L2: Nuclear factor (erythroid-derived 2)-like 2
NF*κ*B p65: Nuclear factor kappa B p65 subunit
NOX: NADPH oxidases (1–4)
P38MAPK: P38 mitogen-activated kinase
PKA: Protein kinase AMPc-dependant
PKC: Protein kinase C
PPARA and G: Peroxisome-proliferator activated receptors alpha and gamma
PPARGC1A: PPARG coactivator 1
PTEN: Phosphatase and tensin homolog
ROS: Reactive oxygen species
RRM2B: Ribonucleotide reductase M2 B (TP53 inducible)
RTCA: Real-time cell analyser
RT-qPCR: Real-time quantitative polymerase chain reaction
SIRT1: Sirtuin 1
SREBP1: Sterol regulatory element binding protein 1
STATs: Signal transducers and activators of transcription
TFAP: Transcription factor AP family
TORC2: CREB regulated transcription coactivator 2
TSC1/2: Tuberous sclerosis 1 and 2
USFs: Upstream transcription factors.

## References

[1] Dey A., Swaminathan K. (2010). Hyperglycemia-induced mitochondrial alterations in liver. *Life Sciences*.

[2] Giacco F., Brownlee M. (2010). Oxidative stress and diabetic complications. *Circulation Research*.

[3] Dentin R., Hedrick S., Xie J., Yates J., Montminy M. (2008). Hepatic glucose sensing via the CREB coactivator CRTC2. *Science*.

[4] Decaux J. F., Marcillat O., Pichard A. L., Henry J., Kahn A. (1991). Glucose-dependent and -independent effect of insulin on gene expression. *The Journal of Biological Chemistry*.

[5] van Deursen D., Jansen H., Verhoeven A. J. M. (2008). Glucose increases hepatic lipase expression in HepG2 liver cells through upregulation of upstream stimulatory factors 1 and 2. *Diabetologia*.

[6] Tu A.-Y., Albers J. J. (2001). Glucose regulates the transcription of human genes relevant to HDL metabolism: responsive elements for peroxisome proliferator-activated receptor are involved in the regulation of phospholipid transfer protein. *Diabetes*.

[7] Iwasaki Y., Kambayashi M., Asai M., Yoshida M., Nigawara T., Hashimoto K. (2007). High glucose alone, as well as in combination with proinflammatory cytokines, stimulates nuclear factor kappa-B-mediated transcription in hepatocytes in vitro. *Journal of Diabetes and Its Complications*.

[8] Sugimoto R., Enjoji M., Kohjima M. (2005). High glucose stimulates hepatic stellate cells to proliferate and to produce collagen through free radical production and activation of mitogen-activated protein kinase. *Liver International*.

[9] Patil M. A., Chua M.-S., Pan K.-H. (2005). An integrated data analysis approach to characterize genes highly expressed in hepatocellular carcinoma. *Oncogene*.

[10] Midorikawa Y., Makuuchi M., Tang W., Aburatani H. (2007). Microarray-based analysis for hepatocellular carcinoma: from gene expression profiling to new challenges. *World Journal of Gastroenterology*.

[11] Teufel A., Weinmann A., Krupp M., Budinger M., Galle P. R. (2007). Genome-wide analysis of factors regulating gene expression in liver. *Gene*.

[12] Berger E., Vega N., Vidal H., Geloën A. (2012). Gene network analysis leads to functional validation of pathways linked to cancer cell growth and survival. *Biotechnology Journal*.

[13] Xu L., Hui L., Wang S. (2001). Expression profiling suggested a regulatory role of liver-enriched transcription factors in human hepatocellular carcinoma. *Cancer Research*.

[14] Wong C. M., Ng I. O. L. (2008). Molecular pathogenesis of hepatocellular carcinoma. *Liver International*.

[15] Huynh H., Nguyen T. T. T., Chow K.-H. P., Tan P. H., Soo K. C., Tran E. (2003). Over-expression of the mitogen-activated protein kinase (MAPK) kinase (MEK)-MAPK in hepatocellular carcinoma: its role in tumor progression and apoptosis. *BMC Gastroenterology*.

[16] Iyoda K., Sasaki Y., Horimoto M. (2003). Involvement of the p38 mitogen-activated protein kinase cascade in hepatocellular carcinoma. *Cancer*.

[17] Nishimura D., Ishikawa H., Matsumoto K. (2006). DHMEQ, a novel NF-*κ*B inhibitor, induces apoptosis and cell-cycle arrest in human hepatoma cells. *International Journal of Oncology*.

[18] Poma P., Notarbartolo M., Labbozzetta M. (2006). Antitumor effects of the novel NF-kappaB inhibitor dehydroxymethylepoxyquinomicin on human hepatic cancer cells: Analysis of synergy with cisplatin and of possible correlation with inhibition of pro-survival genes and IL-6 production. *International Journal of Oncology*.

[19] You M., Matsumoto M., Pacold C. M., Cho W. K., Crabb D. W. (2004). The role of AMP-activated protein kinase in the action of ethanol in the liver. *Gastroenterology*.

[20] Adachi M., Brenner D. A. (2008). High molecular weight adiponectin inhibits proliferation of hepatic stellate cells via activation of adenosine monophosphate-activated protein kinase. *Hepatology*.

[21] Palmeira C. M., Rolo A. P., Berthiaume J., Bjork J. A., Wallace K. B. (2007). Hyperglycemia decreases mitochondrial function: the regulatory role of mitochondrial biogenesis. *Toxicology and Applied Pharmacology*.

[22] Qiao L., MacDougald O. A., Shao J. (2006). CCAAT/enhancer-binding protein *α* mediates induction of hepatic phosphoenolpyruvate carboxykinase by p38 mitogen-activated protein kinase. *The Journal of Biological Chemistry*.

[23] Nishikawa T., Edelstein D., Du X. L. (2000). Normalizing mitochondrial superoxide production blocks three pathways of hyperglycaemic damage. *Nature*.

[24] Díaz-Delfín J., Morales M., Caelles C. (2007). Hypoglycemic action of thiazolidinediones/peroxisome proliferator-activated receptor *γ* by inhibition of the c-Jun NH2-terminal kinase pathway. *Diabetes*.

[25] Berger E., Rome S., Vega N., Ciancia C., Vidal H. (2010). Transcriptome profiling in response to adiponectin in human cancer-derived cells. *Physiological Genomics*.

[26] Benjamini Y., Hochberg Y. (1995). Controlling the false discovery rate: a practical and powerful approach to in multiple testing under depenancy. *Journal of the Royal Statistical Society B*.

[27] Jeong Y.-S., Kim D., Lee Y. S. (2011). Integrated expression profiling and Genome-Wide analysis of ChREBP targets reveals the dual role for ChREBP in Glucose-Regulated gene expression. *PLoS ONE*.

[28] Ruike Y., Ichimura A., Tsuchiya S. (2008). Global correlation analysis for micro-RNA and mRNA expression profiles in human cell lines. *Journal of Human Genetics*.

[29] Sawey E. T., Chanrion M., Cai C. (2011). Identification of a therapeutic strategy targeting amplified FGF19 in liver cancer by oncogenomic screening. *Cancer Cell*.

[30] Dey A., Chandrasekaran K. (2009). Hyperglycemia induced changes in liver: in vivo and in vitro studies. *Current Diabetes Reviews*.

[31] Chiba T., Yokosuka O., Arai M. (2004). Identification of genes up-regulated by histone deacetylase inhibition with cDNA microarray and exploration of epigenetic alterations on hepatoma cells. *Journal of Hepatology*.

[32] Sivertsson L., Ek M., Darnell M., Edebert I., Ingelman-Sundberg M., Neve E. P. A. (2010). CYP3A4 catalytic activity is induced in confluent Huh7 hepatoma cells. *Drug Metabolism and Disposition*.

[33] Cervello M., Bachvarov D., Cusimano A. (2011). COX-2-dependent and COX-2-independent mode of action of celecoxib in human liver cancer cells. *OMICS*.

[34] Foderà D., D'Alessandro N., Cusimano A. (2004). Induction of apoptosis and inhibition of cell growth in human hepatocellular carcinoma cells by COX-2 inhibitors. *Annals of the New York Academy of Sciences*.

[35] Wilkening S., Stahl F., Bader A. (2003). Comparison of primary human hepatocytes and hepatoma cell line Hepg2 with regard to their biotransformation properties. *Drug Metabolism and Disposition*.

[36] Hart S. N., Li Y., Nakamoto K., Subileau E.-A., Steen D., Zhong X.-B. (2010). A comparison of whole genome gene expression profiles of HepaRG cells and HepG2 cells to primary human hepatocytes and human liver tissues. *Drug Metabolism & Disposition*.

[37] Guo L., Dial S., Shi L. (2011). Similarities and differences in the expression of drug-metabolizing enzymes between human hepatic cell lines and primary human hepatocytes. *Drug Metabolism & Disposition*.

[38] Klingmüller U., Bauer A., Bohl S. (2006). Primary mouse hepatocytes for systems biology approaches: a standardized in vitro system for modelling of signal transduction pathways. *IEE Proceedings: Systems Biology*.

[39] Wang B., Hsu S.-H., Frankel W., Ghoshal K., Jacob S. T. (2012). Stat3-mediated activation of microRNA-23a suppresses gluconeogenesis in hepatocellular carcinoma by down-regulating glucose-6-phosphatase and peroxisome proliferator-activated receptor gamma, coactivator 1 alpha. *Hepatology*.

[40] Moran D. M., Mattocks M. A., Cahill P. A., Koniaris L. G., McKillop I. H. (2008). Interleukin-6 mediates G_0_/G_1_ growth arrest in hepatocellular carcinoma through a STAT 3-dependent pathway. *Journal of Surgical Research*.

[41] Kim H., Baumann H. (1999). Dual signaling role of the protein tyrosine phosphatase SHP-2 in regulating expression of acute-phase plasma proteins by interleukin-6 cytokine receptors in hepatic cells. *Molecular and Cellular Biology*.

[42] Hatziapostolou M., Polytarchou C., Aggelidou E. (2011). An HNF4*α*-miRNA inflammatory feedback circuit regulates hepatocellular oncogenesis. *Cell*.

[43] Sun H., Chua M. S., Yang D., Tsalenko A., Peter B. J., So S. (2008). Antibody arrays identify potential diagnostic markers of hepatocellular carcinoma. *Biomarker Insights*.

[44] Miyazaki T., Bub J. D., Uzuki M., Iwamoto Y. (2005). Adiponectin activates c-Jun NH2-terminal kinase and inhibits signal transducer and activator of transcription 3. *Biochemical and Biophysical Research Communications*.

[45] Shaw R. J., Lamia K. A., Vasquez D. (2005). The kinase LKB1 mediates glucose homeostasis in liver and therapeutic effects of metformin. *Science*.

[46] Ma L., Robinson L. N., Towle H. C. (2006). ChREBP·Mlx is the principal mediator of glucose-induced gene expression in the liver. *The Journal of Biological Chemistry*.

[47] Sancho P., Fabregat I. (2011). The NADPH oxidase inhibitor VAS2870 impairs cell growth and enhances TGF-beta-induced apoptosis of liver tumor cells. *Biochemical Pharmacology*.

[48] Marí M., Morales A., Colell A., García-Ruiz C., Fernández-Checa J. C. (2009). Mitochondrial glutathione, a key survival antioxidant. *Antioxidants and Redox Signaling*.

[49] Pajaud J., Kumar S., Rauch C., Morel F., Aninat C. (2012). Regulation of signal transduction by glutathione transferases. *International Journal of Hepatology*.

[50] Morgan K. T., Ni H., Brown H. R. (2002). Application of cDNA microarray technology to *in vitro* toxicology and the selection of genes for a real-time RT-PCR-based screen for oxidative stress in Hep-G2 cells. *Toxicologic Pathology*.

[51] Loiseau D., Morvan D., Chevrollier A. (2009). Mitochondrial bioenergetic background confers a survival advantage to HepG2 cells in response to chemotherapy. *Molecular Carcinogenesis*.

[52] Leclerc I., Kahn A., Doiron B. (1998). The 5'-AMP-activated protein kinase inhibits the transcriptional stimulation by glucose in liver cells, acting through the glucose response complex. *FEBS Letters*.

[53] Rodgers J. T., Lerin C., Haas W., Gygi S. P., Spiegelman B. M., Puigserver P. (2005). Nutrient control of glucose homeostasis through a complex of PGC-1*α* and SIRT1. *Nature*.

[54] Fogarty S., Hardie D. G. (2010). Development of protein kinase activators: AMPK as a target in metabolic disorders and cancer. *Biochimica et Biophysica Acta*.

[55] Nelson L. E., Valentine R. J., Cacicedo J. M., Gauthier M.-S., Ido Y., Ruderman N. B. (2012). A novel inverse relationship between metformin-triggered AMPK-SIRT1 signaling and p53 protein abundance in high glucose-exposed HepG2 cells. *The American Journal of Physiology—Cell Physiology*.

[56] Kah J., Wüstenberg A., Keller A. D. (2012). Selective induction of apoptosis by HMG-CoA reductase inhibitors in hepatoma cells and dependence on p53 expression. *Oncology Reports*.

[57] Nemoto S., Fergusson M. M., Finkel T. (2005). SIRT1 functionally interacts with the metabolic regulator and transcriptional coactivator PGC-1*α*. *The Journal of Biological Chemistry*.

[58] Lu B., Moser A. H., Shigenaga J. K., Feingold K. R., Grunfeld C. (2006). Type II nuclear hormone receptors, coactivator, and target gene repression in adipose tissue in the acute-phase response. *Journal of Lipid Research*.

[59] de Candia P., Blekhman R., Chabot A. E., Oshlack A., Gilad Y. (2008). A combination of genomic approaches reveals the role of FOXO1a in regulating an oxidative stress response pathway. *PLoS ONE*.

[60] Leclerc I., Lenzner C., Gourdon L., Vaulont S., Kahn A., Viollet B. (2001). Hepatocyte nuclear factor-4alpha involved in type 1 maturity-onset diabetes of the young is a novel target of AMP-activated protein kinase. *Diabetes*.

[61] Naiki T., Nagaki M., Shidoji Y. (2002). Analysis of gene expression profile induced by hepatocyte nuclear factor 4alpha in hepatoma cells using an oligonucleotide microarray. *The Journal of Biological Chemistry*.

[62] Hong Y. H., Varanasi U. S., Yang W., Leff T. (2003). AMP-activated protein kinase regulates HNF4*α* transcriptional activity by inhibiting dimer formation and decreasing protein stability. *The Journal of Biological Chemistry*.

[63] Tachibana K., Kobayashi Y., Tanaka T. (2005). Gene expression profiling of potential peroxisome proliferator-activated receptor (PPAR) target genes in human hepatoblastoma cell lines inducibly expressing different PPAR isoforms. *Nuclear Receptor*.

[64] Ba Y., Zhang C.-N., Zhang Y., Zhang C.-Y. (2008). Down-regulation of PGC-1alpha expression in human hepatocellular carcinoma. *Chinese Journal of Oncology*.

[65] Martínez-Jiménez C. P., Gómez-Lechón M. J., Castell J. V., Jover R. (2006). Underexpressed coactivators PGC1*α* and SRC1 impair hepatocyte nuclear factor 4*α* function and promote dedifferentiation in human hepatoma cells. *The Journal of Biological Chemistry*.

[66] Vougier S., Cheung S. H., Li L., Hodgson G., Shaw P. E. (2007). Anomalous behaviour of the STAT3 binding site in the human c-myc P2 promoter. *Biochemical and Biophysical Research Communications*.

[67] Barré B., Avril S., Coqueret O. (2003). Opposite regulation of myc and p21^waf1^ transcription by STAT3 proteins. *The Journal of Biological Chemistry*.

[68] Briata P., Laurino C., Gherzi R. (1989). c-myc gene expression in human cells is controlled by glucose. *Biochemical and Biophysical Research Communications*.

[69] Iyer V. V., Yang H., Ierapetritou M. G., Roth C. M. (2010). Effects of glucose and insulin on HepG2-C3A cell metabolism. *Biotechnology and Bioengineering*.

[70] Polanski M., Anderson N. L. (2007). A list of candidate cancer biomarkers for targeted proteomics. *Biomarker Insights*.

[71] Pirola L., Balcerczyk A., Tothill R. W. (2011). Genome-wide analysis distinguishes hyperglycemia regulated epigenetic signatures of primary vascular cells. *Genome Research*.

